# An Experimental Study of a Composite Wick Structure for Ultra-Thin Flattened Heat Pipes

**DOI:** 10.3390/mi15060764

**Published:** 2024-06-06

**Authors:** Wenjie Zhou, Yong Yang, Junfeng He, Ri Chen, Yue Jian, Dan Shao, Aihua Wu

**Affiliations:** 1 Guangdong Key Laboratory of Battery Safety, Guangzhou Institute of Energy Testing, Guangzhou 511447, China; wuahgz@163.com; 2 School of Mechatronic Engineering, Guangdong Polytechnic Normal University, Guangzhou 510450, China; yy2008@gpnu.edu.cn (Y.Y.); hejunfeng@gpnu.edu.cn (J.H.); rchen01@gpnu.edu.cn (R.C.); jianyue@gpnu.edu.cn (Y.J.)

**Keywords:** heat pipe, wick, filling ratio, thermal performance

## Abstract

As the thickness of an ultra-thin flattened heat pipe (UTHP) decreases, the fabrication difficulty increases exponentially, and the thermal performance deteriorates rapidly. In this study, three types of composite wicks were developed for UTHPs with a 0.6 mm thickness: copper foam and mesh wick (CFMW), two layers of different mesh wick (TDMW), and three layers of the same mesh wick (TSMW). The startup and steady-state performances of the UTHPs with liquid filling ratios of 60% to 120% were investigated. The findings indicated that the CFMW UTHP with a filling ratio of 100% exhibited the best startup performance, with the highest equilibrium temperature of 58.37 °C. The maximum heat transport capacities of the CFMW, TDMW, and TSMW UTHP samples were 9, 8, and 8.5 W, respectively, at their corresponding optimum filling ratios of 110%, 90%, and 100%. The CFMW UTHP exhibited the lowest evaporation and condensation thermal resistances of 0.151 and 0.189 K/W, respectively, which were 24.67% and 41.85% lower than those of the TSMW UTHP. CFMW can be used to improve the thermal performance of UTHPs. This study provides important guidelines for the structural design, fabrication technology, and performance improvement of high-performance UTHPs used in portable electronic devices.

## 1. Introduction

As portable electronic devices have become smaller and more powerful, the operating chips generate more and more heat, making it difficult to dissipate in a timely manner. This results in elevated temperatures within these devices, which in turn constrains their further development [1,2,3]. Ultra-thin flattened heat pipes (UTHPs) exhibit a number of advantages, including small size, lightweight, quick startup performance, good temperature uniformity, and customizable shape [4,5]. Therefore, the rational use of high-performance UTHPs can effectively solve the cooling issue in these devices, reduce the operating temperature, and enhance reliability and safety [6,7]. The heat generated by chips is rapidly transferred to the device’s bracket via UTHPs, and then it is distributed to the surrounding environment through natural convection, thus eliminating the local overheating. Consequently, the thickness and performance of UTHPs are key factors in determining the effectiveness of heat dissipation in devices.

The thickness of a flattened heat pipe is determined by the parameters of the copper tube (e.g., diameter, wall thickness, and length), the manufacturing process, and the application requirements [8]. As the thickness of the UTHP decreases, the difficulty of manufacturing increases exponentially, and the heat transfer performance deteriorates rapidly. Li et al. [9] developed a UTHP with a bilateral arch-shaped sintered wick. The impact of the wick structure, flattened thickness, and particle size on its thermal performance was investigated. The findings indicated that a reduction in the internal cavity results in a notable increase in the flow resistance of the liquid and vapor, accompanied by a corresponding decrease in the thermal performance. Lin and Wong [10] conducted an investigation into the causes of performance degradation in flattened heat pipes. The findings revealed that a liquid accumulation inside the condensation section was the primary factor leading to the deterioration in the performance of the powder-sintered heat pipe, while the main reason for the grooved heat pipe was the reduction in liquid supply to the evaporation section due to the vapor–liquid interfacial shear [11,12], which resulted in the tendency for the heat pipe to dry out. Lurie et al. [13] analyzed a flat heat pipe with a sintered wick using a topology optimization method. The findings demonstrated that the utilization of the optimal wick can enhance its operational performance. In particular, the heat transport capacity was found to be twice that of the heat pipe with a conventional wick.

The wick structure is one of the most critical factors influencing the thermal performance of UTHPs. Yang et al. [14] developed a 1 mm thick UTHP with a copper wire braided wick, which was composed of small-diameter and large-diameter copper wires. Tang et al. [15] studied a novel mesh wick made from copper fine lines. The findings indicated that the wick exhibited a greater capillary force than the conventional wicks. Zhou et al. [16,17,18] developed a variety of spiral woven mesh wick (SWM) structures for use in UTHPs, with the objective of meeting the heat dissipation requirements of a variety of electronic devices. Specifically, a band-shaped SWM structure was designed for UTHPs with a thickness of 0.4 mm to enhance the cooling capacity of smartphones [16]. Additionally, a hybrid SWM wick structure with two diameters of copper wires in strands was fabricated to improve the thermal performance [17], and the effect of the combination of copper wires with two diameters on the thermal performance of UTHPs was studied [18]. The findings indicated that the optimized hybrid SWM wick structure can enhance the heat transport capacity of UTHPs by 53.85%.

The mass of the working fluid exerts a considerable influence on thermal performance. It can be demonstrated that heat pipes with a similar wick structure will only exhibit optimal performance when the liquid filling ratio is appropriate. Li et al. [19] investigated the thermal performance of UTHPs with different filling ratios using a visualization method. The findings showed that a filling ratio of 15% was optimal. If the filling ratio was excessive, the liquid would boil in the evaporator at a higher heating power, and the liquid bridge phenomenon would occur with the liquid subsequently gathering in the condensation section. Chen and Chou [20] designed a flat heat pipe to study the influence of the filling ratio on its thermal performance. The findings indicated that 25% was the optimal filling ratio. Tharayil et al. [21] developed a loop heat pipe to investigate the optimal filling ratio. The experimental findings showed that the optimal value was 30%.

Considering the aforementioned studies, it is evident that numerous scholars have conducted valuable research utilizing theoretical calculations, visualization studies, and experimental analyses. However, there is a paucity of research on the systematic study of UTHP structural design, manufacturing processes, and heat transfer performance. As the thickness of a UTHP decreases, the fabrication of the material and the enhancement of its thermal performance become increasingly challenging. In particular, for thinner UTHPs, the effects of numerous factors, including the material, structure, processes, wick dimension, and working fluid mass, on thermal performance are relatively more significant than those of thicker UTHPs. Consequently, in this study, the manufacturing process and thermal performance of UTHPs with a thickness of 0.6 mm were investigated. Three types of composite wicks were employed in the fabrication of thin-walled cylindrical heat pipes, which were subsequently flattened to manufacture the UTHPs. The startup and steady-state performance levels of the UTHP samples were tested and discussed, with particular consideration given to the effect of heating power, filling ratio, and wick structure. The findings of this study are of significant value in the fields of structural design and fabrication technology regarding performance improvement in high-performance UTHPs, which is a crucial component in portable electronic devices.

## 2. Experimental Samples and Test System

### 2.1. Fabrication of the UTHP Samples

The composite sintered wick structures of the UTHP samples were as follows: copper foam and mesh wick (CFMW), two layers of different mesh wick (TDMW), and three layers of the same mesh wick (TSMW). The three types of composite wicks were constructed using copper foam, 100-mesh, and 200-mesh copper wire meshes, respectively. The thicknesses of the three structures were 0.2, 0.1, and 0.2 mm, respectively. The porosity of the sintered copper foam was 60%. The copper wire meshes were orthogonal woven meshes (OWMs). The CFMW was produced by sintering a layer of copper foam and a layer of 200-mesh copper wire mesh. The TDMW was sintered from a single layer of 100-mesh and a single layer of 200-mesh copper wire mesh. The TSMW was manufactured by sintering three layers of 200-mesh copper wire mesh. Consequently, the thickness of all three composite wicks after sintering was 0.3 mm.

**Figure 1:**
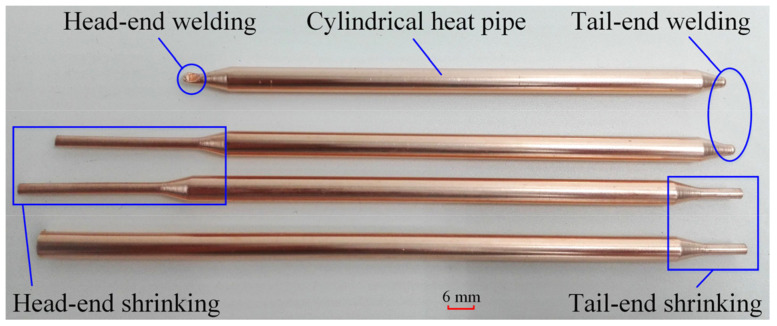
Shrinking and welding processes of cylindrical heat pipes.

Figure 1 illustrates the cylindrical heat pipe following the shrinking and welding processes. The diameter and length of cylindrical heat pipes were 6 and 130 mm, respectively. The UTHP samples were manufactured through a series of processes, including cutting and shrinking the copper tubes, sintering the composite wicks, welding the tail ends, annealing the copper tubes, filling the working fluids, vacuuming and degassing the copper tubes, welding the head ends, and flattening the cylindrical heat pipes. The tube shrinking process for conventional heat pipes was used in the radial forging process (RFP). During the RFP, a three-phase asynchronous motor drives two- or three-split dies in a circumrotation and radial reciprocating movement, while the dies shape the tube with a high-frequency, short-stroke movement. The RFP is generally suitable for the shaping of copper tubes with a thickness of more than 0.20 mm. However, the copper tube thickness for the experimental UTHP was only 0.15 mm, and the strength was found to be poor, particularly after the annealing process. This is because the hard copper tube becomes soft.

The shaping section of the UTHP was susceptible to bending and breaking in the RFP. In order to avoid these serious defects, a novel shrinking process, namely the high-speed spinning process (HSSP), was used to form the experimental copper tube. During the HSSP, a high-speed spindle motor drives an integral die to rotate at a speed of approximately 10,000 rpm, while the die is fed to the tube and the tube is shaped under the synthetic movement of the die. The working area exhibited a high degree of smoothness following the HSSP. The vacuum process was conducted on an automated primary degassing machine. The UTHP samples were vertically inserted into a sealed chamber with a sealing structure that was connected through a tube to a vacuum pump with a limiting vacuum of 0.08 Pa. A digital vacuum gauge was installed on the pipe. As the tube was connected to the internal cavity of the UTHP during the vacuuming process, the vacuum gauge reading could also be used to measure the internal pressure of the UTHP sample. During the actual batch production of UTHP samples, the vacuum gauge reading was 10^−2^–10^1^ Pa, a vacuum level that fully met the requirements of UTHPs.

**Figure 2:**
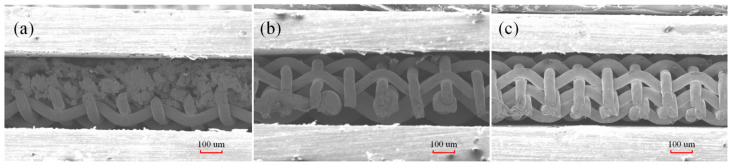
SEM photographs of longitudinal sections of the composite wick structures: (**a**) CFMW, (**b**) TDMW, and (**c**) TSMW.

Following a three-hour sintering process in a high-temperature reducing atmosphere (950 °C, nitrogen–hydrogen mixture gas), the composite wick was attached to the inner wall of the copper tube. As illustrated in Figure 2, scanning electron microscopy (SEM, Zeiss EVO 18 Special Edition from Oberkochen, Germany) was used to examine the microstructures of the three wicks. The samples for SEM observation were prepared by cutting the UTHPs longitudinally with a precision wire-cutting machine. The width and thickness of the CFMW, TDMW, and TSMW were 4.0 and 0.3 mm, respectively. The porosities of the three wicks were measured as 72%, 77%, and 60%, respectively.

**Figure 3:**
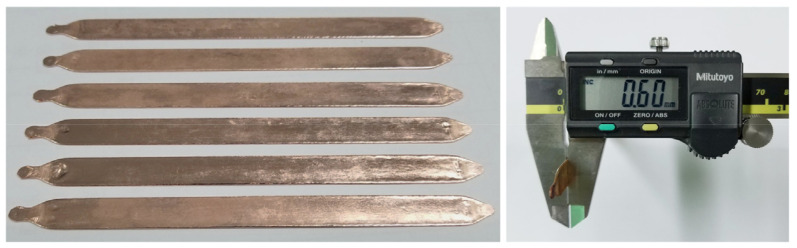
UTHP samples.

The working fluids in the experimental UTHP samples were deionized water with volumetric filling ratios of 60%, 70%, 80%, 90%, 100%, 110%, and 120%. A value of 100% signified that the wick was saturated, indicating that all pores were filled with deionized water. Figure 3 depicts the UTHP samples. The length and thickness of the samples were 130 and 0.6 mm, respectively. The UTHP samples were designated as CFMW UTHP, TDMW UTHP, and TSMW UTHP, in accordance with the wick structures.

### 2.2. Startup Performance Testing of UTHP

**Figure 4:**
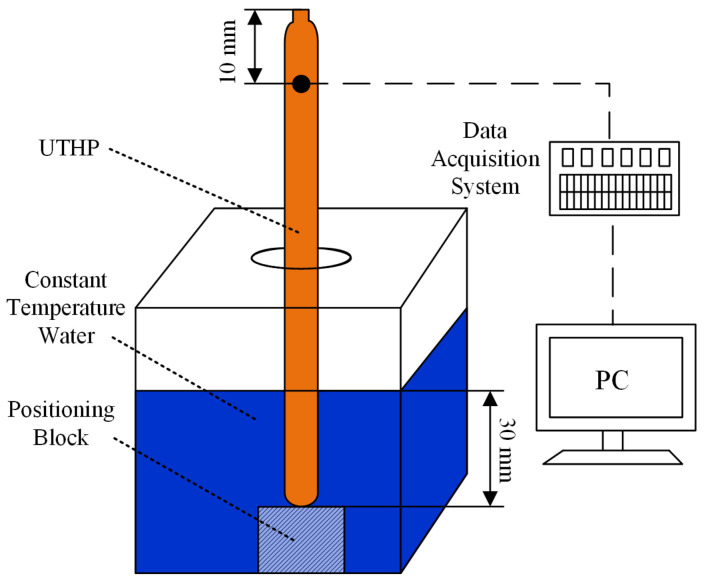
Schematic diagram of the startup performance test system for UTHP samples.

Figure 4 shows the startup performance test system for UTHP samples, which comprised a constant temperature water tank, a positioning block, and a temperature data acquisition system. The sample was positioned vertically during the experiment. The tail end was immersed in the water stored in the tank at a temperature of 60 ± 0.1 °C, with an insertion depth of 30 mm. The head end was maintained in the air. A thermocouple point was established at a distance of 10 mm from the top of the UTHP. The temperature value of the point was collected and recorded using an acquisition system (NI 9213 from Austin, TX, USA). The time from the start of the test to equilibrium was the start time of the UTHP. The equilibrium state was defined as a temperature variation of the point of less than 0.3 °C over a period of 5 s. The ambient environment temperature was 26 ± 1 °C.

### 2.3. Steady-State Performance Testing of UTHP

**Figure 5:**
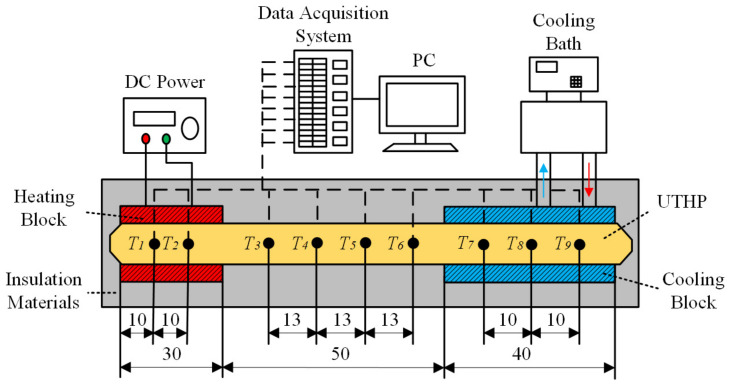
Schematic diagram of the steady-state performance test system (units: mm).

Figure 5 presents the steady-state performance test system for UTHP samples. The system was primarily composed of a heating block, a DC power supply, a cooling block, a water-cooling bath system, and a temperature data acquisition system. A heating rod was inserted into the copper heating block. The heating rod was equipped with a power supply that exhibited an accuracy of 0.5% for current and 0.1% for voltage. The cooling block was cooled by the water at a constant temperature, which was controlled to within a tolerance of 0.2 °C using the water-cooling bath. The water flow rate was regulated with a flow meter with a precision of 2 L per hour. The temperature data were obtained at nine test points by means of T-type thermocouples. Following calibration, the T-type thermocouples had an accuracy of 0.1 °C for temperature. One end of each thermocouple was in contact with the surface of the UTHP, while the other end was connected to an acquisition card with an accuracy of 0.02 °C. During the test, the UTHP sample was positioned horizontally, with its head end and tail end placed on the cooling block and heating block, respectively. The application of thermal grease with a 6.0 W/(m·K) thermal conductivity resulted in a reduction in the contact thermal resistance. A polyurethane block with two test points (*T*_1_ and *T*_2_) was subjected to compression in the evaporation section. The adiabatic section was equipped with four test points, designated *T*_3_, *T*_4_, *T*_5_, and *T*_6_. The condensation section was compressed by the other polyurethane block, which had three test points: *T*_7_, *T*_8_, and *T*_9_. The cooling water was supplied at a rate of 20 L per hour through the cooling block, with a water temperature of approximately 55 °C.

During the test, the UTHPs were insulated with thermal insulation materials to prevent heat loss. The heating power exhibited a range of values between 4.5 and 9 W, with each increment representing a 0.5 W increase. Once the heating power had been adjusted to initiate the test, the test PC recorded the temperature data of all test points until the UTHP sample reached equilibrium. This is defined as a period during which the temperature fluctuation of the points is less than 0.5 °C within 120 s. The maximum heat transport capacity is defined as the maximum heating power. This is the heating power at which the sample continues to operate in a normal state, that is, the evaporation section does not dry out with a rapid rise in temperature, and the temperature difference among the test points is less than 5 °C. 

The average temperatures (*T_e_*, *T_a_*, and *T_c_*) of the evaporation, adiabatic, and condensation sections of a UTHP sample were obtained using the following equations:

(1) $$T_{e}=\frac{1}{2}(T_{1}+T_{2}),$$

(2) $$T_{a}=\frac{1}{4}(T_{3}+T_{4}+T_{5}+T_{6}),$$

(3) $$T_{c}=\frac{1}{3}(T_{7}+T_{8}+T_{9}),$$

The thermal resistances (*R_c_* and *R_e_*) of the condensation and evaporation sections of a UTHP sample were obtained as follows:

(4) $$R_{c}=\frac{T_{a}+T_{c}}{P_{h}},$$

(5) $$R_{e}=\frac{T_{e}+T_{a}}{P_{h}},$$

where *P_h_* is the heating power.

### 2.4. Analysis of the Testing Errors Associated with the Test System

The testing error of the steady-state performance test system is mainly derived from the heating and temperature acquisition system. The relative testing error can be obtained using the following equations [22]:

(6) $$\frac{E[y(x)]}{y(x)}=\frac{\sqrt{\sum_{i=1}^{n}{\left(\frac{\partial y}{\partial x_{i}}E_{x_{i}}\right)}^{2}}}{y(x)},$$

where *x* is an independent variable; *y*(*x*) is a given function of *x*; and *E_x_* and *E*[*y*(*x*)] are the absolute testing errors of *x* and *y*(*x*), respectively. 

According to these calculations, the relative testing error of the heating power was 0.2%, and the maximum relative testing error of the thermal resistance was 6.28%.

## 3. Results and Discussion

### 3.1. Startup Performance of the UTHP Samples

**Figure 6:**
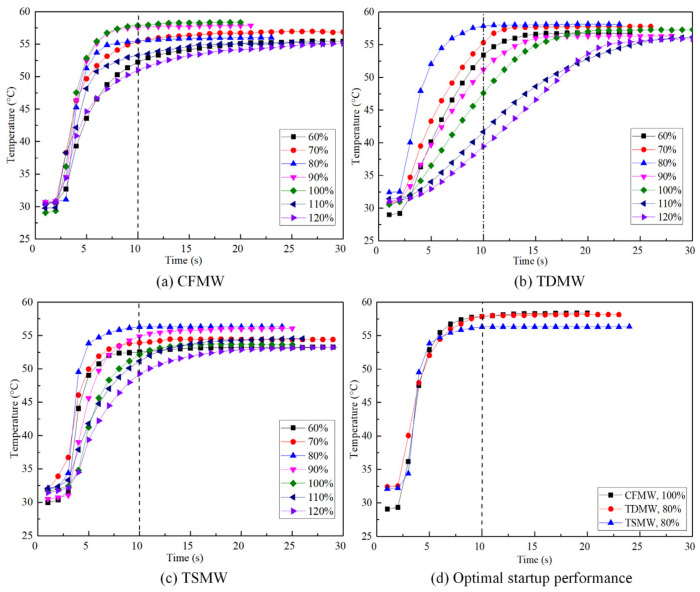
Startup performance of the UTHP samples.

Figure 6 illustrates the startup performance of the UTHP samples with CFMW, TDMW, and TSMW, under varying liquid filling ratios. The temperature of the UTHP test point exhibited a rapid increase and subsequent stabilization with the increase in testing time. The startup performance of the UTHP was also influenced by the changes in the filling ratios. The UTHP samples with CFMW, TDMW, and TSMW demonstrated optimal startup performance at the corresponding fill rates of 100%, 80%, and 80%. This result indicates that the test point temperature increased the most rapidly, while the stable temperature value was the highest. The startup performance of the UTHP samples with CFMW was found to be significantly impaired by high or low filling ratios, but samples with TDMW and TSMW exhibited greater sensitivity to high filling ratios than to low filling ratios. The startup performance of the UTHP samples with TDMW and TSMW deteriorated with an increase in the filling ratio, particularly when the value exceeded 90%. The startup performance of the TDMW UTHP samples was found to be inferior when the filling ratio was 90% in comparison to those with the filling ratios of 60% and 70%. Conversely, the performance of TSMW UTHP was superior when the filling ratio was 90% compared to those with filling ratios of 60% and 70%. For UTHPs, a suitable liquid filling ratio is defined as a sufficient quantity of working liquid present in the evaporation section to participate in the phase change, while the heat source is rapidly dissipated. Therefore, an appropriate filling ratio not only facilitates a rapid startup but also ensures a minimal temperature difference between the evaporation and the condensation sections. Figure 6d illustrates the optimal startup performance of the UTHPs with CFMW, TDMW, and TSMW at 100%, 80%, and 80% filling ratios, respectively. The start times of the three structures were approximately 10 s, and the equilibrium temperatures were 58.37, 58.13, and 56.34 °C, respectively. Among the three structures, the CFMW UTHP exhibited the most optimal startup performance, while that of the TSMW UTHP was the least optimal. The observed performance differences can be attributed to the interconnected nature of the pores inside the CFMW and the minimal backflow resistance of the working liquid. Furthermore, the TDMW and TSMW were composed of orthogonal woven meshes, and the liquid backflow resistance along the direction of their length was considerable. Moreover, the TSMW was sintered by three layers of 200-mesh wires with pores smaller than 100-mesh wires, which resulted in greater resistance.

### 3.2. Temperature Uniformities of the UTHP Samples

**Figure 7:**
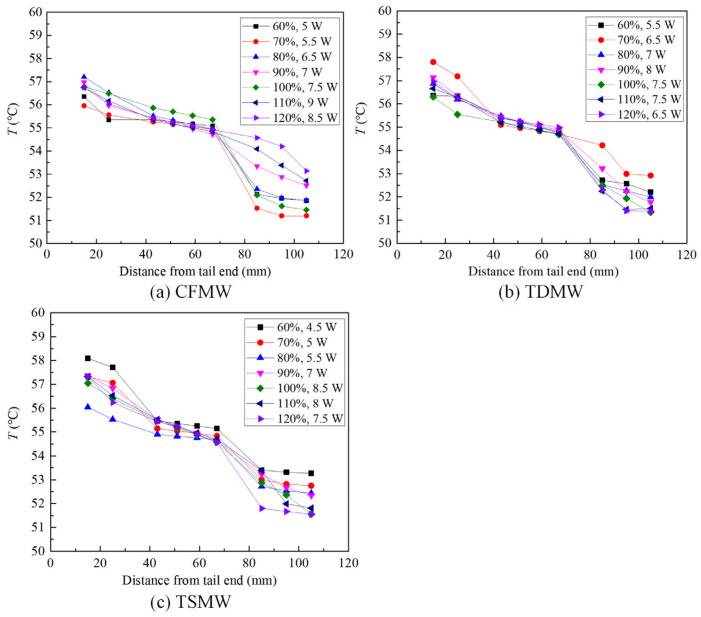
Temperature uniformities of the UTHP samples.

Figure 7 depicts the temperature uniformities of UTHPs with different wick structures and varying filling ratios at their maximum heating powers. The temperature of the evaporation section exhibited a continuous increase in response to an increase in the heat input. These sections were dried out at the heat inputs that exceeded the maximum heat transport capacity (*Q*_max_) values. The UTHP samples with filling ratios below 90% exhibited drying in the evaporation sections with the temperature soaring at a heating power of 7.5 W. This phenomenon could be mitigated if the mass of the working liquid was increased to an appropriate level. However, at a high filling ratio, such as 120%, the liquid would accumulate in the UTHP at a high heating power. This phenomenon would result in a reduction in the effective working length of the sample, accompanied by a shift in the primary mode of heat transfer from phase change to wall conduction. Thus, the heat transfer coefficient was significantly reduced. The performance of the CFMW UTHP exhibited a more pronounced change in response to variations in liquid filling ratios than that of the TDMW and TSMW UTHPs. When the filling ratios were below 80%, the axial temperature differences in the TDMW and TSMW UTHPs were minimal, and these values increased with the heating power. The CFMW UTHP samples demonstrated the optimal overall temperature performance under the appropriate filling ratios in comparison to the TDMW and TSMW UTHPs. The maximum temperature of the evaporation sections was maintained below 57 °C while maintaining a slight difference in temperature. Moreover, the CFMW UTHPs demonstrated the capacity to withstand additional working fluids, which could effectively reduce the dry-out phenomenon. The favorable capillary force of the CFMW facilitated the backflow of the working fluid at high filling ratios. Consequently, the condensation temperature could be maintained above 53 °C at 7.5 W. It was observed that liquid blockages occurred in the condensation sections of the UTHPs with TDMW and TSMW. The temperature differences between the adiabatic and the condensation sections were considerable, with the condensation temperature filling below 52 °C. The *Q*_max_ values were 9, 8, and 8.5 W, respectively, at the corresponding optimum filling ratios of 110%, 90%, and 100% for the CFMW, TDMW, and TSMW UTHPs.

### 3.3. Thermal Resistances of the UTHP Samples

**Figure 8:**
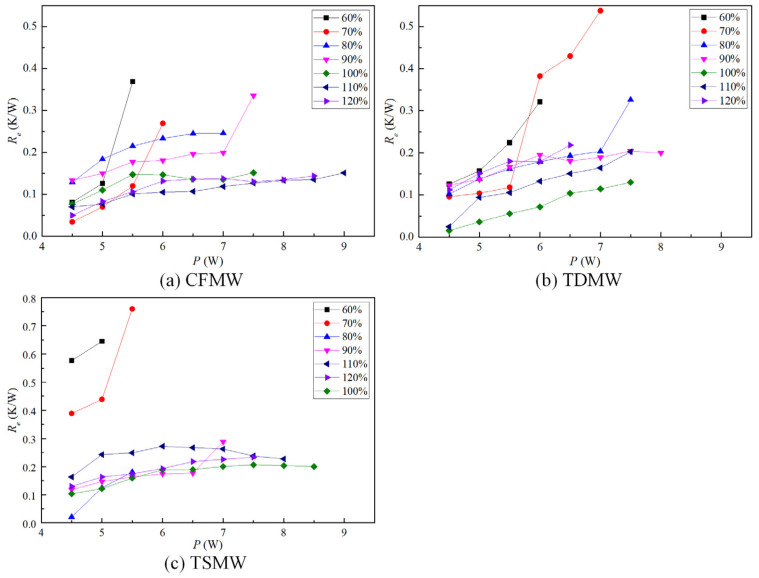
Evaporation thermal resistances of the UTHP samples at various filling ratios.

Figure 8 presents the evaporation thermal resistance (*R_e_*) values of the UTHP samples with CFMW, TDMW, and TSMW at different filling ratios. The overall *R_e_* values in the normal state were found to be stable and below 0.25 K/W. The favorable surface and internal microporous structures of copper foam result in superior capillary properties, which facilitate the replenishment of the evaporation section with working fluid during the phase change process, particularly at high heating powers [23]. Consequently, the CFMW UTHP samples exhibited the lowest *R_e_* of 0.151 K/W. The *R_e_* values exhibited a rapid increase with increasing input power before reaching the dry-out point. The UTHP samples had sufficient working fluid to undergo phase change and absorb additional heat in the evaporation sections, given high liquid filling ratios. Thus, the *R_e_* values exhibited a gradual increase with the input power, maintaining relatively low values. The *R_e_* exhibited a relatively stable profile when the filling ratio was close to the optimal value. The ratio of the wick to vapor spaces was found to be suitable within the UTHP, given the optimal liquid filling ratio. At that time point, neither the phenomenon of drying out nor clogging was observed in the UTHP. The minimal circulation flow resistance of vapor–liquid resulted in relatively low thermal resistance.

**Figure 9:**
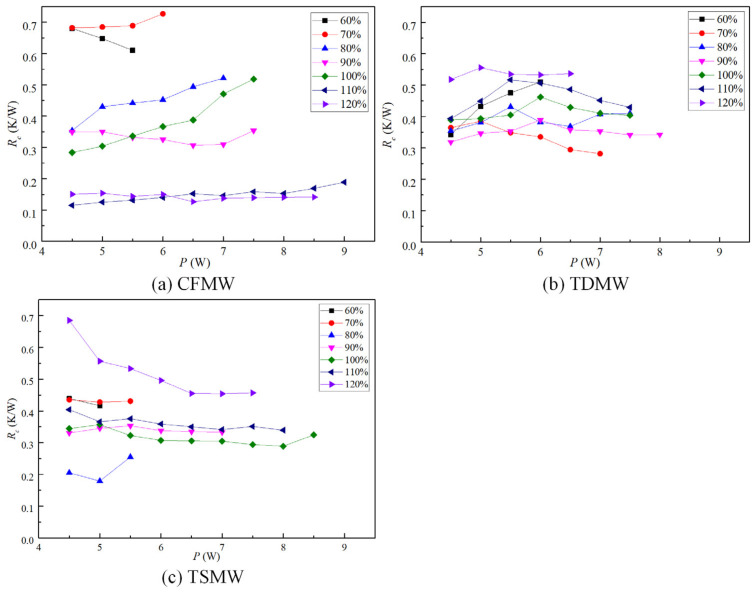
Condensation thermal resistances of the UTHP samples at various filling ratios.

Figure 9 shows the condensation thermal resistance (*R_c_*) values of the UTHPs with CFMW, TDMW, and TSMW at varying filling ratios. The *R_c_* values of the UTHP samples with appropriate filling ratios were relatively stable and below 0.5 K/W. The CFMW UTHP samples exhibited the lowest *R_c_* value, at 0.189 K/W. The CFMW and TSMW UTHP samples were dried out under low liquid filling ratios (60% and 70%). However, under high liquid filling ratios (110% and 120%), the working liquids within the heat pipes became excessive, resulting in the accumulation of liquids in the condensation sections, thereby increasing the *R_c_* values. The *R_c_* values of the CFMW UTHP samples exhibited a significantly lower level at high filling ratios. This reduction was caused by the CFMW, which exhibited a favorable capillary force, effectively enabling the condensation liquid to flow back to the evaporation section to avoid blockage. The *R_c_* values of the TDMW UTHP samples exhibited an initial increase, followed by a subsequent decrease, with increasing input power. The reason is that the heat transfer mass of the UTHP at a low heating power was relatively small, and the blockage of the vapor–liquid flow reduced the condensation temperature and increased the *R_c_* accordingly. As the heating load increased gradually, the temperature difference increased, and the pressure difference between the two ends also increased. The elevated pressure was sufficient to overcome the flow resistance of the working fluid, thus enabling the working fluid to circulate normally and the *R_c_* to gradually decrease.

### 3.4. Comparison of the Maximum Heat Transport Capacity Observed in This Study with Those Reported in Earlier Publications

Table 1 presents the main parameters of the heat pipes with thicknesses ranging from 0.4 to 1.0 mm developed by other researchers and in the present study. The parameters included wick type, total thickness, and *Q*_max_. It can be observed that the *Q*_max_ of the experimental UTHP with a total thickness of 0.6 mm manufactured with the optimized CFMW wick is larger and can reach 9 W, a value that is only slightly lower than the *Q*_max_ of the UTHP with a total thickness of 1.0 mm designed by Tang et al. [24], with a *Q*_max_ value of 11 W. The optimized UTHP sample with CFMW demonstrated good startup performance, reaching a steady state in 10 s during the startup performance test. The temperature at the test point was 58.37 °C, with a temperature difference of only 1.63 °C with the test water. In the steady-state performance test, the CFMW UTHP sample exhibited the lowest evaporation and condensation thermal resistances, with corresponding values of 0.151 and 0.189 K/W, respectively, which were 24.67% and 41.85% lower than those of the TSMW UTHP. This indicates that the CFMW can improve the *Q*_max_ and enhance the thermal performance of the UTHP. The findings of this study provide valuable guidelines for the optimal design of UTHP for cooling portable electronic devices.

## 4. Conclusions

In this study, UTHPs with 0.6 mm thickness were fabricated and investigated for cooling portable electronic devices. The composite sintered wick structures of the UTHP samples were as follows: copper foam and mesh wick (CFMW), two layers of different mesh wick (TDMW), and three layers of the same mesh wick (TSMW). The startup and steady-state performance levels of the UTHP samples with liquid filling ratios of 60% to 120% were tested and studied, with particular consideration given to the effect of heating power, the filling ratio, and the wick structure. The main findings were as follows: 

The filling ratio of UTHP had a greater effect on its thermal performance than that of the wick structure. The startup performance of the CFMW UTHPs was found to be significantly affected by high or low filling ratios. However, the samples with TDMW and TSMW were more sensitive to high filling ratios than to low filling ratios. The startup time of all UTHPs was 10 s, and the equilibrium temperatures were 58.37, 58.13, and 56.34 °C, respectively, at the corresponding filling ratios of 100%, 80%, and 80% for the three types of UTHP samples. The dry-out phenomenon can be alleviated by increasing the mass of the working liquid accordingly. The maximum heat transport capacities were 9, 8, and 8.5 W, respectively, at the corresponding optimum filling ratios of 110%, 90%, and 100% for the three types of UTHP samples. The CFMW UTHP exhibited the lowest evaporation and condensation thermal resistances of 0.151 and 0.189 K/W, respectively, which were 24.67% and 41.85% lower than those of the TSMW UTHP. CFMW can be used to improve the *Q*_max_ and enhance the thermal performance of UTHPs. This study provides important guidelines for the structural design, fabrication technology, and performance improvement of high-performance UTHPs used in portable electronic devices.

## Figures and Tables

**Table 1:** *Q*_max_ value comparison between the present study and earlier publications.

Literature	Wick Type	Total Thickness	*Q*_max_
Chen et al. [25]	OWM and groove	0.4 mm	4.5 W
Zhou et al. [16]	Band-shape SWM	0.4 mm	5.25 W
Yu et al. [26]	Three SWMs	0.4 mm	6 W
Ahamed et al. [27]	SWM	0.6 mm	5 W
Sun et al. [28]	Multi-layer OWM and micropillars	0.67 mm	8.1 W
Cui et al. [29]	Multi-layer OWM	0.68 mm	8 W
Zhou et al. [30]	Center OWM	0.75 mm	8.5 W
Zhou et al. [31]	Copper foam and OWM	0.8 mm	5 W
Tang et al. [24]	Annular OWM	1.0 mm	11 W
Present study	Optimized CFMW	0.6 mm	9 W

## Data Availability

The original contributions presented in the study are included in the article, further inquiries can be directed to the corresponding author.
